# *Erythroxylum* in Focus: An Interdisciplinary Review of an Overlooked Genus

**DOI:** 10.3390/molecules24203788

**Published:** 2019-10-21

**Authors:** David A. Restrepo, Ernesto Saenz, Orlando Adolfo Jara-Muñoz, Iván F. Calixto-Botía, Sioly Rodríguez-Suárez, Pablo Zuleta, Benjamin G. Chavez, Juan A. Sanchez, John C. D’Auria

**Affiliations:** 1 Centro de Estudios sobre Seguridad y Drogas, Facultad de Economía, Universidad de los Andes, Bogota 111711, Colombia; d.restrepod@uniandes.edu.co (D.A.R.); sirodriguezsu@unal.edu.co (S.R.-S.); p.zuleta@uniandes.edu.co (P.Z.); juansanc@uniandes.edu.co (J.A.S.); 2 Departamento Ciencias Biológicas, Facultad de Ciencias, Universidad de los Andes, Bogota 111711, Colombia; je.saenz1181@uniandes.edu.co; 3 Jardín Botánico de Bogotá José Celestino Mutis, Bogota 111071, Colombia; ojara@jbb.gov.co; 4 CEMarin student, Escuela de Biología, Universidad Pedagógica y Tecnológica de Colombia, Tunja 150003, Colombia; ivan.calixto@uptc.edu.co; 5 Department of Molecular Genetics, Leibniz Institute of Plant Genetics and Crop Plant Research (IPK), 06466 Gatersleben, Germany; chavez@ipk-gatersleben.de

**Keywords:** Erythroxylaceae, *Erythroxylum coca*, next generation sequencing, traditional medicine, bioprospecting, tropane

## Abstract

The genus *Erythroxylum* contains species used by indigenous people of South America long before the domestication of plants. Two species, *E. coca* and *E. novogranatense,* have been utilized for thousands of years specifically for their tropane alkaloid content. While abuse of the narcotic cocaine has impacted society on many levels, these species and their wild relatives contain untapped resources for the benefit of mankind in the form of foods, pharmaceuticals, phytotherapeutic products, and other high-value plant-derived metabolites. In this review, we describe the current state of knowledge of members within the genus and the recent advances in the realm of molecular biology and biochemistry.

## 1. Introduction

Humanity has an enduring and intimate relationship with medicinal plants. From prehistoric times through to the modern era, the use of plant specialized metabolites has helped treat diseases, steer religious ceremonies, and cosmetically augment the body. While modern synthetic chemical methods often provide quicker, safer and more cost-effective production of economically important compounds, many plants remain the sole source of such molecules [1,2]. In addition, several plants containing mind altering hallucinogenic or narcotic chemicals have become infamous for the detrimental societal impacts of illicit drugs derived from them [3]. Nonetheless, it is imperative to obtain a deeper understanding of how these plants synthesize their compounds for the future prospects of synthetic biology, metabolic engineering, and potential applications in as far-ranging fields as green medicine and space exploration.

Few plant genera are as rich as *Erythroxylum* L. in their contribution to both highly lucrative legal industries (soft drinks) and one of the largest illicit markets on the planet (cocaine trafficking). The integration of *Erythroxylum* species into the fabric of South American societies makes any discussion of eliminating these plants exceedingly controversial and difficult, with many counterproductive consequences [4]. Indeed, after decades of seemingly fruitless eradication policies, it appears productive and prudent to explore the development of the beneficial uses of these plants, which is an approach that is already being implemented by communities, companies, and governments in South America, within the growing scope of legality provided by the current laws in the Andean region [5]. This approach appears to carry significant potential, given the promising applications indicated by both traditional cultures and the scientific research available. Additionally, recent advances in our knowledge about the biosynthesis of tropane alkaloids, and a growing body of molecular phylogeny studies are revealing a dynamic system of interactions and compounds that justifies further research. Indeed, the use of beneficial products from these plants for medicinal, nutritional, agricultural, and cosmetic purposes is becoming the focus of several countries and scientific groups. In addition, with the ever-increasing capabilities of chemical and genetic analyses, wild species of Erythroxylum are being described and subjected to new studies identifying their potential for future development of medicines. In this review, we consolidate the most recent advances and knowledge in species found within the genus *Erythroxylum*.

## 2. History and Evolution of the *Erythroxylum* Genus

The genus *Erythroxylum* includes approximately 230 species, distributed across the tropics [6]. Two species in this genus, *Erythroxylum coca* Lam. and *Erythroxylum novogranatense* (D. Morris) Hieron., are particularly salient. They have been cultivated for thousands of years by traditional South American societies and, more recently, have become sources for the production of the tropane alkaloid cocaine (commonly diverted into the global illicit trade). 

Each one of these two species commonly referred to as “coca” can be subdivided into two regionally and phenotypically defined varieties, *E. coca* var. *ipadu* Plowman and *E. novogranatense* var. *truxillense* (Rusby) Plowman. The typical variety of *E. coca* is also known as Huánuco or Bolivian Coca (*E. coca* var. *coca*). According to Plowman (1986) [7] this variety probably originated from the eastern Andes of Perú and Bolivia, particularly in the Huállaga Valley, where wild coca plants can be found. The typical altitude of coca crops in this region is between 500 and 1500 m above sea level, but reaching 2000 m in some areas [7]. *E. coca* var. *coca* is the most common cultivar used in cocaine production for the drug trade in Perú and Colombia. Amazonian coca, or Ipadú coca (*E. coca* var. *ipadu*) is grown in the Amazonian region, which includes Colombia, Brazil, and Perú. Colombian coca (*E. novogranatense* var. *novogranatense*) is cultivated in the inter-Andean valleys of Colombia, and in the Sierra Nevada de Santa Marta region. It can also be found as a domestic garden plant in urban settings. Finally, Trujillo coca (*E. novogranatense* var. *truxillense*), which grows mainly in northern Perú, and is the variety best adapted to the dry, arid climates of Perú’s coastal deserts. 

Coca is among the oldest cultivated medicinal plant species, with evidence of its use dating back at least 8000 years in South America [8,9]. Traditional coca consumption remains prevalent across scores of Andean and Amazonian communities. In 2012, Bolivia’s traditional coca-using population was estimated at around three million people [10], while in 2013, Perú’s coca-using population numbered around 3.5 million [11]. In addition, significant traditional coca-using populations are also found in Argentina, Brazil, Chile, and Colombia, although their numbers are unknown [12].

Coca leaves contain the highest amounts of cocaine and other tropanes in the plant [13]. The total alkaloid content of coca leaves varies by variety and climate. For example, Bolivian coca contains on average 0.63% dry weight cocaine in the leaves [14], whereas, *E. coca* var. ipadu (Amazonian coca) contains between 0.11% and 0.41% cocaine content. No significant differences were witnessed between laboratory and field grown Amazonian coca plants, suggesting that the biosynthesis of tropane alkaloids is regulated at the molecular genetic level. The highest cocaine levels identified in common cultivars of coca are obtained from the *E. novogranatense* varieties. In this species, cocaine levels vary from an average of 0.77% in Colombian coca to 0.72% in Trujillo coca. A study by Acock et al. (1996) [15] revealed that while light intensity (photosynthetic photon flux density) did not seem to have much of an effect on alkaloid production, temperature did have an effect. Lower temperatures seemed to adversely affect alkaloid content in all studied cultivars. 

Coca leaves are traditionally consumed by forming a quid in the cheek (i.e., in English this is called “coca chewing”, although little chewing is involved). Frequently, the coca leaf quid is complemented with a basified material or alkaline adjuvants. These are generally processed (usually burnt) from diverse sources such as plant ashes (i.e., quinoa, tree leaves, and cinnamon), animal bones, seashells, mineral deposits, and sodium bicarbonate. These alkaline adjuvants bear a multitude of names across South America (i.e., cal, llipta, ilucta, mambe, tocra, lejía, etc.) [16]. The mode of traditional coca consumption varies according to the coca variety and the alkaline substance used. For example, Huánuco coca leaves are chewed directly following the addition of the alkaline powder (cal), which is alternated with additional leaves and cal. The alkaline powder is often substituted with sodium bicarbonate or other adjuvants, especially for non-native consumers. Colombian coca and Trujillo coca are both chewed with ashes made from incinerated seashells, bones, or mineral deposits found in limestone rock formations. Amazonian coca is processed more extensively, presumably with the aim of strengthening the release of its lower alkaloid content, via a complex process that includes the mild roasting and powdering of the leaves, followed by filtering with a fine mesh or fabric and mixing with dried leaf ashes of the species *Cecropia sciadophylla* Mart. Other species of the Cecropiaceae, such as *Cecropia peltata* L. and *Pouruma cecropiifolia* Mart. are occasionally used when *C. sciadophylla* is unavailable [7,17]. Schultes (1981) [17] makes reference to the addition of other plants to the coca powder to increase its effects or improve flavor. For example, several species within the genus *Costus* L., one species of *Styrax* L., and the palms *Cocratea exorrhiza* (Mart.) H.Wendl. and *Astrocaryum gynacanthum* Mart. *(=Astrocaryum munbaca* Mart.) have all been documented for this purpose. The subjective effect on human consumers is described as energizing and mood enhancing, as well as providing a sense of well-being, while suppressing feelings of appetite and thirst [18].

Coca has a rich social, cultural and medicinal significance in the traditional South American cultures where it is cultivated and used [12]. It is employed, disproportionately by adult men, to promote individual work performance and self-discipline as well as to strengthen community ties via work-related social gatherings, “town hall” style meetings, religious ceremonies, and important life events (such as weddings and funerals), where sharing and using coca are central activities [19,20,21]. Coca also bears metaphysical significance, as it is used as an offering to nature, in divinatory practices, and as part of rituals believed to help sustain the balance between the human and natural worlds [19]. 

In traditional medicine, coca is utilized as a remedy for a wide variety of conditions, ranging from alleviating oral pains, digestive maladies, hunger, altitude sickness, muscular and skeletal aches, as well as sadness and sexual impotence [22]. The uses and potential of coca in traditional and contemporary medicine, nutrition, and agriculture are further explored in Section 4. 

### 2.1. The Phylogeny of Erythroxylum and Related Genera 

The families Erythroxylaceae and Rhizophoraceae make up a well-supported clade in the order Malpighiales [23]. Both families share numerous morphological characteristics [24], as well as specific types of alkaloids that are rare in other groups of angiosperms, such as hygroline, tropane alkaloids, and pyrrolizidines [25]. In the Erythroxylaceae, *Erythroxylum* is placed adjacent to the clade formed by the African genera *Nectaropetalum* Engl., *Pinacopodium* Exell and Mendonça, and *Aneulophus* Benth. The clade formed by these three genera is sister to the genus *Aneulophus* Benth., also endemic to Africa [26]. A phylogenetic framework of *Erythroxylum* has been recently reported by Islam (2011) [27] and White et al. (2019) [28]. This work provides a greater understanding of the subgeneric relationships within the genus and complements the original monograph written over 100 years ago [29]. Recent phylogenetic studies agree with earlier work, suggesting that most of the sections proposed by Schulz (1907) [29] are not monophyletic [14,30,31,32].

**Figure 1:**
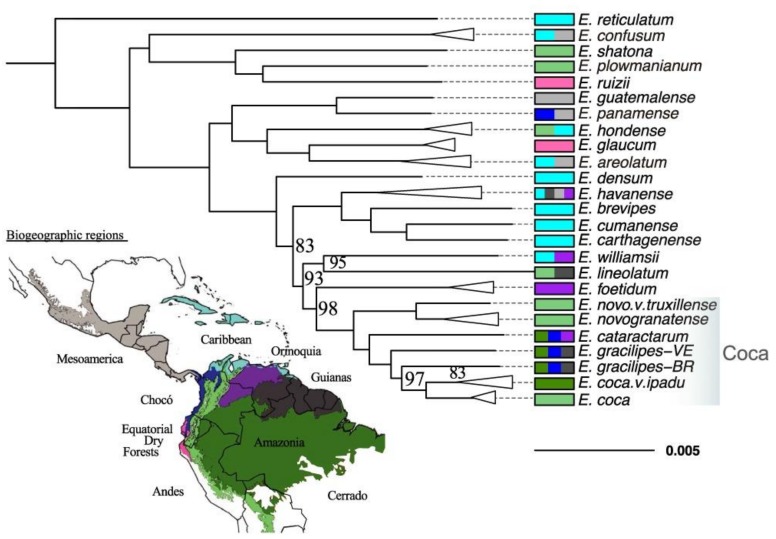
Phylogeny of the *Erythroxylum* genus. Colors on the map and the bars next to the names indicate the biogeographic regions where the species are naturally distributed. Numbers on the nodes indicate bootstrap support, which is 100% where not indicated. Modification of the phylogeny, inferred by White et al. (2019) [28] in the American Journal of Botany, 106(1), p. 158, is republished with permission from the Botanical Society of America.

The phylogeny presented by Islam (2011) [27] is based on two loci, the intergenic spacer of the chloroplast rpL32-trnL, and the intergenic spacer of the Ribosome (ITS), whereas the tree presented by White et al. (2019) [28] is based on 547 nuclear genes. These studies are consistent with each other when considering the position of the cultivated species. In both phylogenies, *E. novogranatense* and *E. coca* are not sister species, contradicting the results from Bohm et al. (1982) [33]. According to White et al. (2019) [28], cultivated species stay in a clade with approximately 24 species (see Figure 1), distributed mainly in the northwestern region of the Neotropics, which in turn includes a mainly Caribbean clade, with species such as, *Erythroxylum brevipes* DC., *Erythroxylum carthagenense* Jacq., *Erythroxylum cumanense* Jacq., and *Erythroxylum havanense* Jacq., and a mainly Amazonian and Andean clade which includes the two cultivated species, as well as *Erythroxylum cataractarum* Spruce ex Peyr., and *Erythroxylum gracilipes* Peyr.

*Erythroxylum* species, with current or potential biomedical applications, belong to a variety of clades within the genus’s phylogeny. The American species are grouped in sections of *Erythroxylum, Archerythroxylum,* and *Macrocalyx*. *Archerythroxylum* is a paraphyletic grouping, containing at least seven American sections [28]. *Macrocalyx* is polyphyletic and nested within the *Archerythroxylum* species [30]. Species belonging to *Archerythroxylum* include *Erythroxylum vacciniifolium* Mart. which is located in clade I (sensu [28]), *Erythroxylum caatingae* Plowman which is a sister species in clade V (also containing the cultivated species), and *Erythroxylum ovaliifolium* Peyr. whose phylogenetic location is unknown. *Erythroxylum subsessile* (Mart.) O.E. Schulz belongs to *Erythroxylum* and is located also in clade I. *Erythroxylum suberosum* A.St.-Hill., A.Juss. and Cambess has not been included in molecular phylogenies, but there is morphological and anatomical evidence to place it near *Erythroxylum macrophyllum* var. *savannarum* Plowman in clade III [30].

Few African species have been included in phylogenetic analyses, however, it can be inferred from White et al. (2019) [28] that these species do not constitute a monophyletic grouping. At least one of these groups, which includes *Erythroxylum nitidulum* Baker, is sister to the rest of the genus. The African species with biomedical applications belongs in two sections as follows: *Erythroxylum macrocarpum* O.E. Schulz and *Erythroxylum laurifolium* Lam. can be found in the *Pachylobus* section and *Erythroxylum pervillei* Baill. belongs to the *Eurysepalum* section. Finally, the Asian *Erythroxylum cuneatum* (Miq.) belongs to the *Coelocarpus* section, which is morphologically diverse and possibly not monophyletic, with species present in Australia, Southeast Asia, and West Africa. 

Most *Erythroxylum* species are distyle, featuring individuals whose flowers have short styles and long stamens (brevistyles) and long styles and short stamens (longistyles). This characteristic was first described by Darwin [34]. Such floral dimorphism is associated with self-incompatibility in the genus, which has been shown experimentally to be stronger in *E. coca* than in *E. novogranatense* [33,35]. Crossing has been successful between *E. novogranatense* varieties, whereas crossing is only successful between species when one of the individuals is a staminated *E. coca*. Crosses are much more frequent when they involve *E. novogranatense* var. *truxillense* [33]. 

In studies of wild and distyle species in the genus, it has been shown that *Erythroxylum havanense* Jacq. is self-incompatible [36], while *Erythroxylum amazonicum* Peyr. is self-compatible [37]. More detailed *E. havanense* analyses have proven the existence of sharp pollen fertility differences across flower types, suggesting an active evolutionary transition toward dioicity [38,39,40]. A similar loss of the distyle condition, expressed as seed production without pollination (agamospermy), was detected for *Erythroxylum undulatum* Plowman at the embryological level [41]. 

### 2.2. Domestication of the Coca Plant

The first explicit hypothesis about coca domestication was based mainly on the results of hybridization experiments and flavonoid profiles of the cultivated varieties [33]. Under this hypothesis, Bohm et al. (1982) [33] proposed a linear process of domestication of the cultivated varieties. This postulated series of domestication events started with *E. coca*, which is thought to have originated from a wild relative, presumably in the Amazonian foothills of Perú and Bolivia. In this domestication model, the Amazonian variety Ipadú originated from the Andean-adapted species. Trujillo Coca was then developed independently as an adaptation to the dryer conditions of the northern regions of Perú. Finally, Colombian coca was selected from Trujillo coca, with an adaptation to the wetter environments of the inter-Andean valleys of Colombia.

Phylogenies based on amplified fragment length polymorphism (AFLP) fingerprinting [42,43], support the monophyly of each cultivated species, *E. coca* and *E. novogranatense*. Johnson et al. (2005) [43] reported that both cultivated species are genetically well separated and have similar intraspecific variability, which is a pattern that contradicts the linear hypothesis of domestication. This genetic structure pattern, combined with the allopatric distribution of both species, suggests an independent origin, which makes both species sister groups. At the intraspecific level, AFLP data showed little variation in *E. coca* var. *ipadu*, which is consistent with the clonal mode of propagation in this variety [9,44,45], and with the domestication hypothesis of Bohm et al. (1982) [33]. Genetic distance between varieties of *E. novogranatense* was negligible, however, because differences in ecological preferences and leaf flavonoid chemotypes are clearly distinguished, taxonomic status of these varieties was not rejected [46,47,48,49].

The most robust taxonomically-sampled phylogenies produced by Islam et al. (2011) [27] and White et al. (2019) [28] indicate that the cultivated species of coca are not sister groups, contradicting the results of the studies mentioned above. According to White et al. (2019) [28] *E. coca* is located in a clade that includes *E. gracilipes* (paraphyletic) and *E. cataractarum* (Figure 1)*,* while *E. novogranatense* is the sister species of the clade formed by these three species. Under this scenario, White et al. (2019) [28], proposed a new hypothesis, with independent domestication events, one of these from *E. cataractarum* to produce *E. novogranatense*, and the second one from *E. gracilipes* to produce *E. coca*. This view is supported by the phylogenetic position of the taxa, and by the geographical distribution of the potential ancestor species, with *E. cataractarum* closer to *E. novogranatense*, and *E. gracilipes* closer to *E. coca*. More genetic evidence at the population level could help produce a more conclusive hypothesis of coca domestication. In addition, improving the taxonomic sampling in section *Archerythroxylum* would also help resolve the question of the proper placement of species in this clade.

### 2.3. Omic Studies on Erythroxylum to Shed Light on Evolution and Functional Background 

Next-generation sequencing (NGS) technologies have revolutionized the acquisition of genetic information and the understanding of molecular processes. Recovering high-throughput genetic data in *Erythroxylum* can provide an integral perspective regarding key components of its genome. For instance, this data can uncover the basis of current diversification patterns by shedding light on the functional meaning (i.e., the function of DNA sequences), structural organization (including the genetic mapping and sequencing of entire chromosomes or genomes), and the scale of genomic differences (such as constructing multiple genome alignments to assess major evolutionary events such as chromosomal rearrangements). Population and phylogenetic studies are migrating from traditional molecular markers such as microsatellites and AFLP [42,43], to screening methodologies coupled to NGS platforms. To date, few efforts have been reported regarding the use of omic data for *Erythroxylum* species. Noteworthy exceptions are the Sanger library of sequenced cDNA and the 454 transcriptome data both made from biosynthetically active young leaf tissue [50,51], which characterize the biosynthesis of alkaloids. In addition, there is a reported chloroplast genome for *E. novogranatense* (NCBI accession number NC_030601.1), produced from whole genome sequencing using a short-read high-throughput strategy via an Illumina platform. Finally, there is the previously mentioned study of White et al. (2019) [28] across *Acherythroxylum* members, which utilized a gene-target sequencing strategy (exome sequencing) and a draft genome assembly for *E. coca* as a reference for phylogenetic purposes. 

A cost-effective strategy to obtain genomic information is reduced representation sequencing (RRS), where thousands of single nucleotide polymorphisms (SNP) are homogeneously screened with genotype neutral loci and adaptive loci detected as outliers from the genomic background (for extended literature see [52,53,54]). Neutral loci can provide a measure of genetic differentiation to address several questions in demography, phylogeography, or population genetics based on how the genetic diversity is partitioned across individuals, populations, and species in *Erythroxylum*. In addition to supporting our knowledge of evolutionary processes, these types of studies can provide new approaches for tracing gene flow dynamics, allowing the development of marker-based tools. These can help the identification of new cultigens, as well as the loci involved in the biosynthesis of compounds of interest (such as alkaloids). 

Omics data also have the potential to produce useful information for several bioprospecting targets in *Erythroxylum*, such as the ones referenced in the next two chapters. This information can serve as a platform for gene discovery, the development of breeding programs (where phenotypic traits are linked to specific sites or genes, using strategies such as marker assisted selection, MAS), and even the development of pharmaceutical products using gene editing tools. Nevertheless, limited published data on these efforts is available, particularly in comparison to other species that produce specialized metabolites and are also used as illicit substances. The most conspicuous example is the research on *Cannabis*, which is at least a decade ahead in available omic information (genomes, transcriptomes, genetic maps, metabolic pathways, etc.) and is already succeeding in using this data to generate desirable plant characteristics for industrial purposes. This includes the development of plant lines where the biosynthesis of tetrahydrocannabinol (THC, the molecule producing psychoactive effects) is minimized, and cannabidiol (CBD, with broad medical applications) biosynthesis is enhanced [55,56], boosting a growing pharmaceutical and phytotherapeutic market. 

*Cannabis* research demonstrates the importance of modern omics technologies in deciphering and making productive use of genetic resources. Within the bioprospection studies in *Erythroxylum*, such endeavors involve reconstructing the overall metabolic network of tropane biosynthesis, where association studies between particular phenotypes of *E. coca* and *E. novogranatense* are coupled with whole genomes (both assembled and annotated), transcriptional profiles, SNP screens (for putative genes of interest), and whole metabolomic data. This type of biological information could serve as a knowledge platform with multiple productive applications. These range from detailing the history and evolution of *Erythroxylum* species to identifying the metabolic pathways (as well as promoters and inhibitors) of key compounds (such as alkaloids and flavonoids) to enabling more effective crop management strategies. 

## 3. An Overview of Bioprospection and Pharmacological Research in the *Erythroxylum* Genus 

The widespread distribution of the *Erythroxylum* genus has led its member species to face diverse climates, herbivore pressures, and soil nutrient conditions, resulting in a wide array of adaptations. These include the evolution of multiple general and specialized metabolites with nutritional and biomedical potential. Such a large reservoir of molecules offers an opportunity for the use of up-to-date “screening” tools that can help identify the productive applications of this genus across many biochemical pathways. Table 1 summarizes the applications of *Erythroxylum* metabolites found across the health literature. 

### 3.1. Erythroxylum vacciniifolium *Mart.*

**Figure 2:**
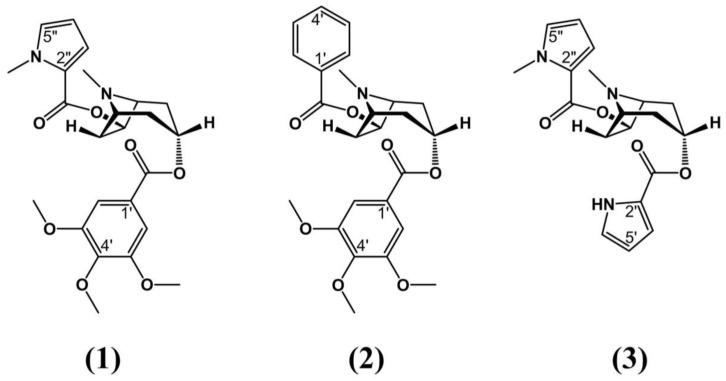
Example of novel tropane alkaloids found in extracts of *E. vacciniifolium.* Structural data was determined using high-resolution electrospray ion cyclotron resonance mass spectroscopic analysis [57].

*E. vacciniifolium* is a popular medicinal plant in Brazil. An infusion made with this plant is called “catuaba” (a name also applied to infusions of plants belonging to other genera). *E. vacciniifolium* stands out for having tonic and aphrodisiac properties. According to Zanolari et al. (2003) [57], these effects are linked to the plant’s tropane content, including catuabines **A**, **B**, and **C**. Extensive research has been conducted on *E. vacciniifolium* and other species in this genus to identify bioactive compounds with medicinal properties. These bioactive properties are linked to the compounds’ chemical structure, such as the presence of C-3 α ester, a moiety not often found in tropanes of the Erythroxylaceae. For catuabines **A** and **B**, this is C-3 3,4,5 trimethoxybenzoic acid and for catuabine C it is pyrrole-2-carboxylic acid [58]. Novel tropane alkaloids are shown in Figure 2.

**Figure 3:**
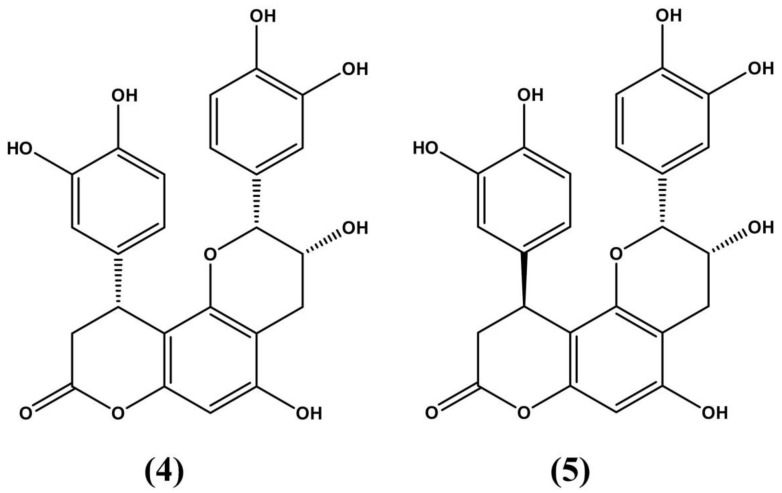
Cinchonains 1a (**4)** and 1b (**5**) as reported in Satoh et al. (2000) [60].

Indigenous people utilize the leaves of the plant as a stimulant. In addition, its bark is used as a remedy for erectile dysfunction. Alkaline extracts and teas made with *E. vacciniifolium* appear to act on the human immunodeficiency virus (HIV) and against opportunistic infections [57]. This may stem from the antimicrobial activity of the *E. vacciniiifolium* extract against *Escherichia coli* and *Staphylococcus aureus*, which can cause lethal infections especially in patients with compromised immune systems [59]. Catuaba’s cytotoxic activity has also been studied, as it yields two cytotoxic flavonoids, cinchonains [DR1] 1a (**4**) and 1b (**5**). Applying these flavonoids to L1210 mouse leukemia cell cultures results in a significant reduction in the number of cancerous cells. [60] (Figure 3).

### 3.2. Erythroxylum ovalifolium *Peyr.*

**Figure 4:**
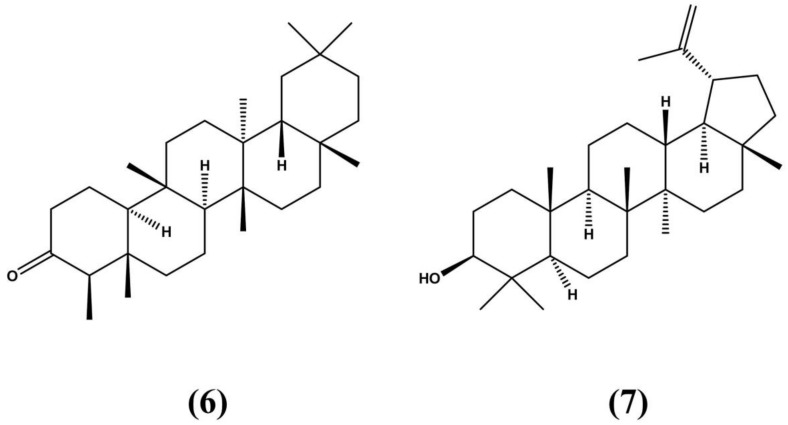
The triterpenoids friedelin (**6**) and lupeol (**7**) isolated and described in *E. ovalifolium* extracts [61].

*E. ovalifolium* is a woody shrub extensively distributed along Brazil’s coastal plains. As with other species in the genus, this shrub has attracted research interest due to its medicinal bioactive properties. *E. ovalifolium* extracts can significantly neutralize the toxic effects of snake venom, such as the one produced by the Southern American bushmaster *Lachesis muta.* Additionally, these extracts also minimize edemas and hemorrhages, symptoms associated with snake venom exposure. Pure *E. ovalifolium* extracts have been noted for their antifungal properties against fibrous fungi such as *Fusarium guttiforme* and *Chalara paradoxa.* Along with other species in the genus, *E. ovalifolium* can produce tropane alkaloids. Medicinal triterpenoids, such as friedelin (**6**) and lupeol (**7**) Figure 4, and a host of flavonoids have also been detected in *E. ovalifolium* extracts [61]. 

### 3.3. Erythroxylum pervillei *Baill.*

*E. pervillei* stands out in its genus for having the most applications across both biotechnology and medicine. Several isolated compounds from *E. pervillei* are used to counteract drug resistance in tumor-based diseases [62].

**Figure 5:**
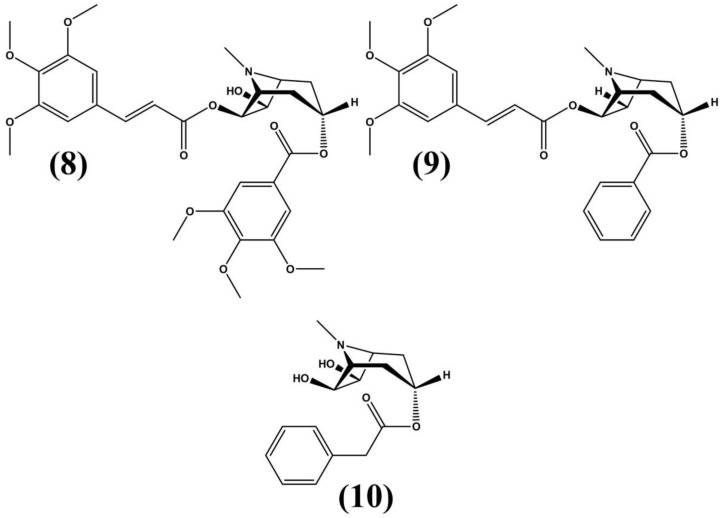
Pervilleine A (**8**) and other aromatic esters isolated from extracts of *E. pervillei* [64].

For instance, pervilleines such as pervilleine A (**8**) with *N*-oxide are used to revert pharmacological resistance in small tumor panels. These compounds are known for their trimethoxycinnamate group at C-6, which confers the cytotoxic and antitumor properties commonly found in these plants. Additionally, these compounds restore vinblastine and colchicine sensitivity in several cell lines. Furthermore, methanolic extracts and tropane alkaloids from *E. pervillei* preparations are used in traditional medicine for their cytotoxic properties and as antineoplastic agents, inhibiting malignant tumor growth [52]. In traditional medicine, *E. pervillei* preparations are used for their cytotoxic properties and as antineoplastic agents, inhibiting malignant tumor growth. Traditional communities also use *E. pervillei* roots for treating abdominal pain. Among certain cultures, this plant is known as “Tsivano” and is employed as a fish poison [63,64]. This research resulted in the identification of novel aromatic esters shown in Figure 5.

### 3.4. Erythroxylum macrocarpum *0.E. Schulz*

Several studies have produced descriptions of *E. macrocarpum*’s components and medicinal properties. The plant is rich in tannins, phenols, flavonoids, and alkaloids, with the latter displaying antimicrobial activity. Aqueous extracts of *E. macrocarpum* feature a wide spectrum antibiotic activity against test organisms like *Staphylococcus aureus,* however, there are no reports of antifungal properties associated with these extracts. It is noteworthy that most of the antibacterial compounds are found in the plant’s leaves, and less frequently in branches or roots [65].

**Figure 6:**
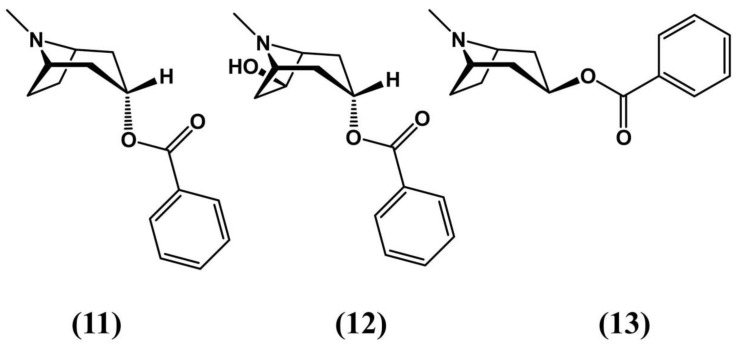
Examples of tropanes found in *Erythroxylum macrocarpum* [67].

Several studies suggest that *E. macrocarpum* displays a notable diuretic effect (i.e., it augments the flow of urine), which may have clinical value in several kidney disorders. This activity is achieved by means of compounds that limit the Na+ permeability of enterocytes, reducing the electrochemical gradient and minimizing the force that propels fluid through the small intestine. In this manner, the liquid reabsorption is inhibited across the nephron’s proximate, distal and collector ducts, which leads to the production of high urine quantities [66]. The plant’s main alkaloids are benzoyl esters of tropan-3α-ol, tropan-3β-ol and tropan-3α, 6β-diol, as well as their Nor-derivates, which contribute to this plant’s medicinal characteristics [67]. Examples of these structures can be found in Figure 6.

### 3.5. Erythroxylum caatingae *Plowman*

*E. caatingae*, another species found in Brazil was reported to have antifungal and antimicrobial activity with little cytotoxic activity in mice [63]. In particular, the tropane catuabine B, 6β-benzoyloxy-3α-(3,4,5-trimethoxybenzoyloxy), elucidated by De Oliveira et al. (2011) [68], induced a rise in early apoptosis in cells from 53.0% to 74.8% [63]. 

### 3.6. Erythroxylum Suberosum *A.St.-Hill., A.Juss and Cambess*

*E. suberosum* is a small and woody shrub popular in Brazil as a medicinal plant. It is claimed to have anti-diarrhea, astringent, anti-rheumatoid, and anesthetic properties among others. There has been little research to ascertain the chemistry behind these effects. Nonetheless, Ribeiro et al. 2015 [69] highlights some of the properties of *E. suberosum* extracts and suggests the presence of alkaloids, coumarins, flavonoids, anthocyanins, tannins, and tri-terpenes as causal agents. He also mentions that the *E. suberosum* extract displays high antioxidant effects through DPPH reduction, mediated by the phenolic compounds from this plant [69]. This last claim is confirmed in Barros et al. 2017 [70], wherein he isolates isoquercitrin, quercetin, catechin, and epicatechin isomers displaying antioxidant activities. Additionally, extracts from this plant show cytotoxic activity from these compounds in head and neck cancers, particularly tongue and hypopharynx carcinomas [71]. 

### 3.7. Erythroxylum laurifolium *Lam.*

Found in the Mauricio region in Brazil, this plant is thought to have antidiabetic medicinal properties. One mechanistic study reported the plant’s inhibitory effect on important carbohydrate hydrolysis enzymes, including amylase and α-glucosidase. Additionally, the plant’s extracts appear to trap glucose via its kinetic effect on amylosis. These extracts show stronger medicinal effects if prepared with methanol rather than water [72]. Ethanol extracts from *E. laurifolium* inhibit angiotensin enzymes, an effect used in treating arterial hypertension from heart and kidney failure. The effect is linked to *E. laurifolium*’s proanthocyanidins or condensed tannins and flavonoids quercitrin and afzelin [73]. Additionally, its oligomeric and polymeric proanthocyanidins act against the Herpes simplex type I virus, by disrupting its replication via enzyme inhibition [74]. Similarly, the plant extracts’ antimicrobial activity has been evaluated against *Staphylococcus aureus*, *Escherichia coli*, *Pseudomonas aeruginosa,* and *Salmonella typhi*. These effects are attributed to the isolated tannins found in *E. laurifolium* extracts [75].

## 4. *Erythroxylum coca* and *E. novogranatense*: Coca’s Productive Uses 

### 4.1. Context of Coca’s Uses 

Coca’s traditional use as a natural stimulant among native South Americans prompted a boom in scientific interest during the 19th and early 20th century. This history is extensively researched by Gootemberg (2008) [76]. This initial scientific interest led to the isolation of cocaine and its derivatives, the first anesthetics identified by science, contributing to the rise of modern anesthetic-assisted surgery and pharmaceuticals, as well as the emergence of the soft-beverage industry (including Coca Cola, which still contains decocainized coca leaf extracts). 

Nevertheless, the early 20th century backlash against cocaine, due to its toxicity and potential for addiction, drove the stigmatization of whole coca [76] and has held back research and development activity regarding its potential applications. Indeed, legal hurdles, stigma, and conflation with cocaine, compounded by the lack of awareness regarding coca’s unique characteristics, have impeded research on whole coca and its non-cocaine components [77,78]. To this day, the science regarding whole coca’s risk profile and productive applications remains limited, both due to the paucity of research on this topic and the need to replicate and enhance existing studies [79]. For instance, the available articles on coca’s physiological and health effects (referenced throughout this section) consist of case studies rather than clinical trials. This is unsurprising given the obstacles to performing coca research thus far. That said, traditional medicine, the incipient research available on coca, and the findings from related *Erythroxylum* species indicate that there is potential for addressing the coca research gap and developing coca’s productive applications.

### 4.2. Whole Coca versus Isolated Cocaine 

Coca’s safety profile, due to its cocaine content, is a key consideration when pondering the development of coca’s productive applications. The Biondich and Joslin (2016) [79] review explores coca’s safety profile and finds several factors that may contribute to the safety of whole coca leaf products as compared to the health risks associated with cocaine isolates. First, whole coca leaf products expose humans to significantly lower cocaine content. Whole leaves average 0.1% to 0.9% cocaine weight and, typically, traditional coca chewers consume 60 g of leaf over the span of a day, resulting in gradual, partial absorption of coca’s alkaloids. Secondly, because the cocaine present in whole leaf is not as readily absorbed as cocaine isolates (particularly, the soluble cocaine hydrochloride salt), peak cocaine concentrations in the blood are approximately 50 times lower than when cocaine isolates are consumed. Third, whole coca is believed to contain three endogenous alkaloids, as well as yield some 17 other alkaloids [80,81], belonging to the tropanes, pyrrolidines, and pyridines. According to Novák et al. (1984) [82], coca’s other alkaloids are significantly less toxic and active than cocaine and, based on Rubio et al. (2015) [83], at least some of these are also significantly absorbed. Potentially, these alkaloids interact with similar receptors as cocaine and may contribute to different, and possibly milder, pharmacological outcomes. 

A topic that has not received significant scientific attention is the pharmacokinetic activity of whole coca alkaloids. As whole coca’s endogenous cocaine and related alkaloids are not stabilized as a cocaine salt (i.e., cocaine hydrochloride), whole coca’s endogenous cocaine may react in the presence of saliva and alkali solutions in the oral mucus. This could result in the partial breakdown of cocaine into other alkaloids that also contribute to different pharmacological outcomes vis-à-vis cocaine isolates. Similarly, the presence of other phytochemicals (such as flavonoids) with significant metabolic activity is poorly documented and their effects and interactions with coca’s other components remains unexamined in the literature. 

Nersesyan et al. (2013) [84] produced a case study of whole coca’s oral cancer risk and found indications that it was potentially lower than other psychoactive plants used orally. Nersesyan et al. (2013) [84] detected no nuclear DNA damage resulting from coca use, however, they noted some acute cytotoxicity when coca is accompanied with alkaline adjuvants [84]. The extent to which these effects may or may not increase cancer risk is unknown. 

The multiple factors that reduce coca’s risks vis-à-vis isolated cocaine may explain the absence of reports regarding whole coca harms. The WHO/UNICRI Cocaine Project 1995 [85], which allegedly reviewed coca and cocaine, could not identify evidence of negative health consequences for coca leaf chewing or whole coca product formats [85], however, this review went unpublished due to political pressures and was only recovered for general dissemination after lobbying efforts by civil society organizations [86]. It should be noted that this WHO project did not conduct extensive epidemiological research on coca leaf consumption, which remains a gap in the scientific evidence.

### 4.3. Potential Uses in Contemporary Medicine 

**Figure 7:**
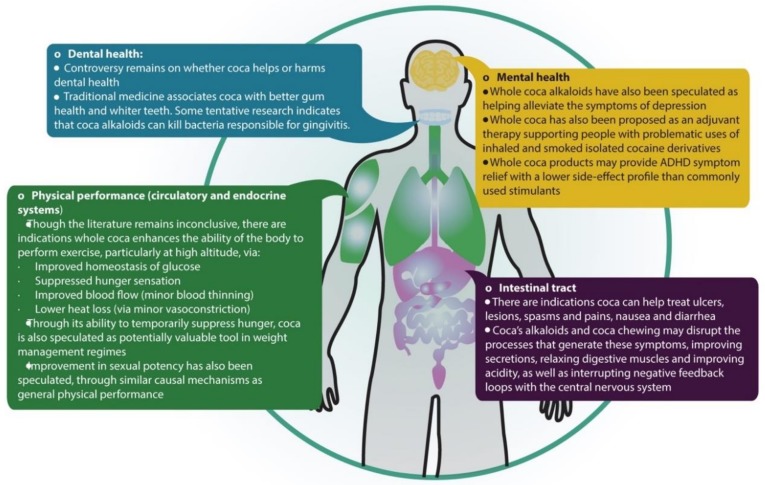
Potential biomedical uses of the coca plant across mental health, dental health, physical performance, and intestinal tract.

The potential value of coca leaf in contemporary medicine is hypothesized based on both traditional medicine reports and the small number of studies that have managed to overcome the legal constraints, logistic barriers, and stigma surrounding this plant. As mentioned earlier, the literature is limited in volume and constitutes a low level of evidence across all of coca’s applications but may indicate a significant opportunity to use updated techniques exploring hypotheses about coca. Additionally, more work is needed to explore the molecules and interactions behind the physiological effects observed (Figure 7). Though coca’s alkaloids may well drive many of the observations, other chemical families (such as flavonoids) could play an important part as well, however, a full characterization of coca’s alkaloid and nutrient content with contemporary techniques remains absent and thus hinders further research studies in this area.

It is worth noting that some of the available research on whole coca does not appear to account for the challenge that novice coca chewers may encounter in mastering the techniques of traditional coca consumption. This may limit research participants’ ability to fully realize coca’s effects. Anthropological reports point out that learning these techniques takes time, even among members of traditional cultures [16,19]. Future research may require methods or product formats that facilitate product adoption to assess the full effects of coca and the variation of these effects among novice and experienced users. 

#### 4.3.1. Coca and Physical Performance: Metabolic and Cardiovascular Effects

Among the areas of interest for contemporary medicine, a key topic explored in the literature has been coca’s value as a stimulant and its effect on physical performance, which is the application most akin to coca’s traditional use. 

The available literature explores several whole coca effects on the endocrine and vascular systems, associated with physical performance enhancement. First, there is the presumed increase in glucose availability, especially during physical exertion [87,88,89,90,91,92], which may be achieved via coca’s effects on promoting fatty acid metabolism [93]. Secondly, Weil’s (1981) [22] review identified a subjectively reported temporary appetite suppression, which may be linked to coca’s promotion of higher glucose availability. Third, there are hypotheses that coca use is associated with improved blood flow and reduced heat loss [94,95], possibly connected with mild vasoconstriction, higher hemoglobin levels, and blood thinning effects [96]. These may stem from coca’s alkaloids atropine-like behavior, that temporarily reduces the rate of red blood cell production, resulting in lower blood viscosity [97].

Overall, coca’s physical performance effects are relatively mild, but potentially clinically significant. Biondich and Joslin (2015) [98] and Biondich and Joslin (2016) [79] indicated that coca’s impact on glucose availability appears to be coca’s most scientifically-validated metabolic effect [93]. They reported the value of using coca in reducing the symptoms of altitude sickness. The effects on glucose metabolism may provide a basis for the appetite suppression that Weil (1981) [22] hypothesizes could support coca’s use in weight management regimes, as well as, potentially, diabetes management. Coca’s blood thinning effects, speculated in Fuchs (1978) [97], may also contribute to the low incidence of thrombosis in native Bolivian populations, as noted by Rodriguez (1997) [96]. This would indicate the potential of coca products for stroke prevention, as long as coca’s vasoconstrictive effects are properly accounted for and managed.

#### 4.3.2. Coca and Digestive and Oral Health

Weil (1981) [22] reported coca’s digestive health application in traditional Andean medicine, where it was used for alleviating gastric tract ulcers, lesions, spasms and pains, nausea, and diarrhea. It can be hypothesized that coca’s tropane alkaloids may employ similar metabolic pathways as hyoscine, with proven clinical value in managing digestive symptoms. Montesinos (1965) [99] and Weil (1981) [22] speculated that coca’s anesthetic alkaloids may disrupt the negative feedback loops between the central nervous system and the digestive tract that generate these symptoms, thereby improving secretions, relaxing digestive muscles and regulating acidity.

Traditional Andean cultures ascribe positive dental effects to coca, claiming it whitens teeth, improves gum health, and treats tooth aches, oral infections, and sores [22], however, the potentially corrosive effect on tooth enamel of the lime often used as an adjuvant in coca chewing may undermine this effect. Indeed, archeological analysis in Odin (1996) [100] indicates that ancient coca chewing populations displayed worse dental health than their non-coca chewing counterparts, although other factors, such as differences in diet, may have played an important role. Initial case studies indicated that coca extracts kill the main bacteria responsible for gingivitis [101] and has general antiseptic effects [102]. Less corrosive alkaline adjuvants, such as sodium bicarbonate and calcium carbonate from ash, are also common in many coca cultures [16] and may prevent dental harm. To settle this controversy, additional research is required on coca’s composition (especially tooth-staining tannin content), its antimicrobial effects, and the role of alkaline adjuvants. 

#### 4.3.3. Sexual Impotence

Though there is no confirmatory research on coca’s impact on sexual performance, there are ample anecdotal and documented reports claiming coca’s value for this application [103,104]. The improvement in sexual performance may be linked to coca’s effect on glucose metabolism, mood, and blood flow linked to its alkaloid, flavonoid, and nutrient content. In any case, aphrodisiac properties, such as improved erectile function, have also been claimed for congener species like *E. vacciniifolium* [57].

#### 4.3.4. Mental Health and Problematic Drug Use

There are initial proposals for coca’s role in providing tools for several important mental health conditions. Weil (1981) [22] indicated that coca may act as a fast-acting antidepressant, owing to the mood-enhancing effects of its main alkaloids. This would imply the potential value of integrating whole coca products into depression treatment pathways. In terms of attention deficit and hyperactivity disorder (ADHD), it can be hypothesized that coca may provide analogous clinical benefits to current treatments available. As whole coca contains several stimulants in the tropane family, whole coca products and isolated alkaloids could well provide similar outcomes to those achieved by methylphenidate and amphetamines, which are currently used in ADHD management [105], however, it may be necessary to determine whether coca and coca alkaloids act on both dopamine and noradrenaline neuron receptors, which are key targets in the pharmacological treatment of ADHD [106]. 

Finally, Hurtado Gumucio (1995) [107] reported the potential value in using coca leaf products to treat addictions to stimulants. This study featured a case study with 50 subjects displaying problematic use of insufflated cocaine hydrochloride and smoked cocaine sulphates (coca paste). Subjects were given psychotherapeutic support and whole coca products as part of a harm reduction strategy aimed at improving participants’ social functionality. The case study indicated significantly higher scores on social functionality measures after interventions using whole coca products.

### 4.4. Potential Uses in Nutrition 

Several studies have provided insight into coca’s comparatively high nutritional contents [108,109,110]. Particularly, coca leaf contains significant quantities of protein, carbohydrates, fiber, minerals (especially calcium, phosphorus, and iron), and vitamins, such as thiamine, riboflavin, and carotene [108,109,111]. When consumed as tea, coca provides minerals such as calcium, magnesium, potassium, iron, manganese, zinc, phosphorus, copper, sulfur, sodium, and aluminum. Potentially harmful minerals are found in such low quantities that they do not appear to pose a health risk [110].

Despite coca’s high nutritional density, Penny et al. (2009) [111] questioned coca nutrient bioavailability, due to the presence of absorption inhibitors common across green vegetables and leaves like coca. Although research on bioavailability of coca minerals and protein is limited, Collazos, Uriquieta, and Alvistur (1965) [108], one of the few reported clinical studies, provides an initial basis for assessing the bioavailability of coca’s vitamins. It found that coca chewing extracted 100% of thiamine, 37% of riboflavin, and 62% of carotene available in the leaf. Additionally, absorption capacity can be improved via certain additives. Extrapolating from Hallberg and Huthén (2000) [112], ascorbic acid can be used as an additive to promote the absorption of plant minerals, such as iron, and this strategy may be applicable to whole coca-based products. 

Although coca’s nutritional contents are insufficient for a whole diet, they may be valuable in dietary supplementation [109,110]. These may be particularly relevant in traditional and indigenous communities in South America, where malnutrition remains a concern. To fully establish the potential of whole coca products as a dietary supplement, it would also be necessary to confirm the low risk of coca’s alkaloid contents and their effect on nutritional outcomes [111].

### 4.5. Potential Uses in Agriculture

There are reports of coca’s potential for organic fertilizers, animal feed, and pesticides. The case for using coca for plant and animal nutrition is based on its high macro- and micronutrient contents, particularly its significant amounts of vegetable protein. Several rural initiatives for turning coca leaf into organic fertilizers are mentioned in the media across the Andean region. Coca-based fertilizers are proposed as a strategy for rural communities to improve local food production and reduce fertilization costs [113]. In terms of animal feed, a rodent case study found coca protein to be less nutritious than cow’s milk protein, but of sufficient quality to provide adequate rodent nutrition [114]. This contrasts with research cited in Penny et al. (2009) [111], which observed that rodents did not gain sufficient weight and showed liver abnormalities when offered whole coca-based diets. Further work on coca animal feed is required to settle the controversy. Finally, Nathanson et al. (1993) [115] claimed coca alkaloids act as a pesticide with insecticidal effects at naturally-occurring concentrations. This indicates that coca-based insecticidal sprays may provide crop protection against pests.

### 4.6. Legality and Development of the Coca Industry in the Andean Region and Beyond

The coca plant and ecgonine-bearing *Erythroxylum* species are prohibited globally for cultivation, transformation, and consumption under the UN’s international drug control regime [77], despite their potential for productive applications. The only exemptions are medical and scientific uses of coca, as well as decocainized coca leaf extracts (utilized by Coca Cola) [76]. However, the main coca growing countries, Bolivia, Colombia, and Perú, have invoked international laws guaranteeing indigenous people their right to defend and promote their cultural practices and have thereby created a space of legality for coca products [116]. Indeed, fully legal markets for whole coca products have operated in Bolivia and Perú for decades [117], while in Colombia these markets operate in a more tenuous grey area [77]. In all three countries, the space of legality coexists alongside eradication and crop substitution policies targeting coca farmers and aimed at curtailing coca cultivation for the trade in illicit cocaine. The legitimacy of these policies is questioned, as they are associated with exacerbating violence and causing social, public health, and environmental harms. These policies are also considered ineffective, as they focus a disproportionate amount of resources on the least lucrative and most readily replaceable section of the illicit cocaine supply chain [118]. 

Despite the legal barriers, numerous formal and informal companies have emerged across the Andean region selling whole coca products, across food and personal care categories. These are registered by or operate through government agencies in Perú and Bolivia [12,117,119] or are licensed via indigenous authorities in Colombia [77]. In terms of food products, coca is sold as whole dried leaf, pulverized leaf, or as an infusion. It is also used as an ingredient in soft drinks, alcoholic beverages, breads, pastries, and confectionery (including coca chocolates and sweets). There are also a variety of personal care products, such toothpastes, gels and ointments in which coca is used as an ingredient. 

Licit and illicit coca market data are patchy, however, the data available indicate coca represents a significant agricultural market that impacts a sizeable population. The United Nations Office on Drugs and Crime reported the 2017 coca crop in the Andean region covered 245,500 hectares [120]. On the basis of Colombian and Peruvian data [121], lot sizes per coca-growing household are estimated at roughly one hectare. This means that perhaps a quarter of a million households across the Andean region are engaged in coca farming. The UNODC estimated that the total 2017 Andean coca crop yielded about one million metric tons of leaf. With average leaf prices per kilo of about USD 2, the coca harvest in the Andean region generated some USD 2 billion in revenue (UNODC 2019) [120]. This is significant for the region’s agricultural economies. In Bolivia alone, for instance, the coca harvest is estimated at USD 375 to 461 million and may represent 8% to 10% of its agricultural gross domestic product [122]. 

Much coca leaf today is processed into illicit cocaine. In Perú, it is estimated that over 90% of coca harvested is directed into the illicit market [12]. In Colombia, the share of the illicit market is likely even higher, as its traditional coca consuming population is thought to be vastly smaller than either Perú’s or Bolivia’s. In contrast, the data available for Bolivia indicate a smaller share of the illicit cocaine market there, as 19,000 tons of Bolivia’s coca leaf is said to be used for traditional consumption out of a total 35,500 to 44,200 metric tons produced [122]. 

Once the coca leaf from across the Andean region is processed into cocaine, it yields nearly 2000 metric tons [120], estimated to reach a global black market value of USD 94 to 143 billion once it is shipped around the world [123]. This represents an enormous source of illicit profits linked to crime, violence, and environmental degradation in the Andean region and beyond. 

Increasing the research and innovation of coca’s applications could, therefore, constitute a high-impact social, economic, and environmental opportunity. For instance, if the evidence of coca’s safety could be scientifically established, it would be viable to strengthen and extend legal supply chains for coca products both in the Andean region and internationally. Indeed, scientific research and innovation on nutritional, medicinal, and agricultural coca products could help coca compete as a less harmful alternative to current illicit stimulants, while increasing the size of coca’s legal market opportunity. Overall, this would help divert money away from the illicit drug trade, while generating legal economic options for coca farmers. 

## 5. Inclusive and Equitable Research and Commercialization of *Erythroxylum* Species 

To advance the coca research agenda, it is important to note that the main coca growing countries, Bolivia, Colombia, and Perú, are all party to the 2010 Nagoya Protocol. This international legal framework sets out to prevent undue appropriation and exploitation of cultural and genetic resources, while attempting to ensure benefits are fairly distributed among stakeholders, particularly, indigenous and traditional small farmer communities from which they are derived [124]. Each country has laid out its own approaches to comply with the Nagoya Protocol commitments, including community consultation mechanisms, collective branding, and appellation of origin, among others. In addition to legal compliance, there are ethical considerations in ensuring indigenous and small farmer communities stand to gain significantly from coca research activities. Not only have these communities experienced a disproportionate amount of the burden created by the policies against coca cultivation [118], but they have also safeguarded coca cultural knowledge and practices [76], making it possible for science to continue conducting research on these plants. It is, therefore, both a legal and ethical priority for research and commercialization models involving species in the genus *Erythroxylum* to enable the active participation, leadership, and fair benefit sharing of indigenous and farmer communities. 

## 6. Tropane Alkaloid Biosynthesis in *E. coca*

Early research into the pharmaceutically active components of the coca leaf began in the mid 1800s with the first description and crystallization of cocaine [125]. The original pioneering research in tropane alkaloid biosynthesis was performed by the chemist Edward Leete, starting in the early 1960s. During this early period, the main methods used to elucidate intermediates in the biosynthetic pathway relied upon the use of feeding radiolabeled potential precursors to whole plants, followed by chemical degradation analyses [126,127]. In a variety of tropane producing plants, [2-^14^C] ornithine feeding has produced conflicting results. A symmetrical incorporation of ornithine is reported for *Erythroxylum coca*, whereas unsymmetrical incorporation is evident in solanaceous plants [128,129,130,131,132]. Ornithine and arginine are converted into putrescine directly by either ornithine decarboxylase or indirectly by arginine decarboxylase, agmatine iminohydrolase, and *N*-carbamoylputrescine amidohydrolase, respectively [133]. Remote isotope labelling of *N*-methylputrescine shows stereoselective incorporation into the first ring in both *E. coca* and multiple species from the Solanaceae [134,135]. The committed step in tropane alkaloid biosynthesis in solanaceous plants is the formation of *N*-methylputrescine catalyzed by an *S*-adenosyl-L-methionine (SAM) dependent methyltransferase. The responsible enzyme was first isolated from tobacco [136] and other orthologs have been isolated from both the Solanaceae and the Convolvulaceae [137,138]. The [6-^14^C] spermidine feeding to solanaceous plants led to the symmetrical distribution of radioactivity of the first ring, however, no further experiments were performed that investigated spermidine as the main polyamine involved [139]. The first ring closure occurs spontaneously from 4-methylaminobutanal to form the *N*-methyl-Δ^1^-pyrrolinium cation [140]. This ring structure was shown to be incorporated into the final bicyclic alkaloid cocaine by feeding labeled [2-^14^C]-1-methyl-Δ^1^-pyrrolinium chloride and [1-^13^C, ^14^C, ^15^N]-4methylaminobutanal diethyl acetate to intact *E. coca* plants [141].

Many hypotheses have been formed as to the origins of the second ring closure in tropane alkaloids. For many years, the compound hygrine was thought to be a direct intermediate based on feeding studies, but more recent studies using stable isotopes demonstrate that previous results are artifactual [142,143,144]. In solanaceous plants the best incorporation into the second ring has been achieved from racemic ethyl [2,3-^13^C_2_]-4-(*N*-methyl-2-pyrrolidinyl)-3-oxobutaboate [142,145]. Further evidence is demonstrated by the feeding of methyl (RS)-[1,2-^13^C_2_,1-^14^C]-4-(1-methyl-2pyrrolidinyl)-3-oxobutanoate to the leaves of *Erythroxylum coca* [146]. This strongly implicates the involvement of acetate derived metabolites in the formation of the second ring in tropane alkaloids.

Historically, the sole sources of data regarding enzymes involved in tropane alkaloid biosynthesis were derived from studies of members in the Solanaceae, however, a recent report has identified and characterized ornithine and arginine decarboxylases in *Erythroxylum coca*. The catalytic activity of ODC was confirmed through a yeast ODC deficient spe1Δ mutant complementation with EcODC. The spe1Δ mutant transformed with EcODC showed 80 times more spermidine than the untransformed spe1Δ mutant [50]. The committed step in tropane alkaloid biosynthesis is thought to be the formation of *N*-methylputrescine catalyzed by an S-adenosyl-L-methionine (SAM) dependent putrescine methyltransferase (PMT). Oxidative deamination of *N*-methylputrescine by methylputrescine oxidase (MPO) gives rise to a reactive intermediate (4-methylaminobutanal) which is thought to spontaneously cyclize, generating a five-membered ring, the *N*-methyl-Δ^1^-pyrrolinium cation. In solanaceous species, the MPO belongs to a copper-dependent class of diamine oxidases [147,148].

To date, there have not been any reported ring closure enzymes characterized for tropane alkaloids in *Erythroxylum coca,* however, in *Atropa belladonna*, the formation of the tropane ring is catalyzed by two enzymes. First, a non-canonical type III polyketide synthase (AbPYKS) utilizes the *N*-methyl-Δ^1^-pyrrolinium cation as a starter substrate and undergoes two rounds of malonyl-CoA-mediated chain elongation to yield 4-(1-methyl-2-pyrrolidinyl)-3-oxobutanoic acid. Then the 4-(1-methyl-2-pyrrolidinyl)-2-oxobutanoic acid undergoes a cyclization reaction mediated by tropinone synthase (AbCYP82M3), a cytochrome p450 enzyme to form tropinone [149]. The tropane biosynthetic pathway in coca differs from the pathway in solanaceous plants in that the decarboxylation at the 2-position does not occur and instead the carboxy group is methyl esterified to form 2-carboxymethyl-3-tropinone (methylecgonone). No information regarding the protection and retention of this function is available, however, the discovery of methyl salicylate in the essential oil of coca leaves suggests that carboxymethyl transferases are active in this tissue [150]. It is clear, however, that formation of this second ring results in the production of a bicyclic alkaloid containing an α-keto group at position C-3. While the biosynthetic pathway of tropane alkaloids in *E. coca* has varied from *A. belladonna* it would be reasonable for homologous enzymatic reactions to be shared in the formation of the tropane alkaloid backbone.

**Figure 8:**
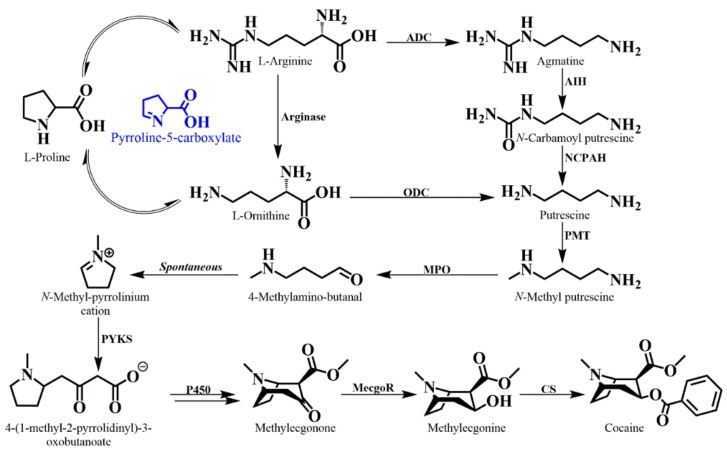
The theoretical tropane alkaloid biosynthetic pathway in *E. coca*. The name of each enzyme, along with their respective acronyms, and corresponding GenBank accession numbers are described. Enzymes with GenBank accession numbers have been reported and characterized. Enzymes without GenBank accession numbers have not yet been described in *E. coca*. The following enzymes are depicted in the figure above: Arginase, arginine decarboxylase (ADC) (accession no. JF909553), ornithine decarboxylase (ODC) (accession no. JF909554), agmatine iminohydrolase AIH, *N*-carbamoylputrescine amidohydrolase (NCPAH), putrescine methyltransferase (PMT), *N*-methylputrescine oxidase (MPO), pyrrolidine ketide synthase (PYKS), cytochrome p450 (p450), methylecgonone reductase (MecgoR) (accession no. GU562618), and cocaine synthase (CS) (accession no. KC140149).

Reduction of the keto group is catalyzed by a member of the aldo-keto reductase family in *E. coca* which is in contrast with the use of a short chain dehydrogenase/reductase enzyme used by members of the Solanaceae [138,151]. This finding supports the theory that the ability to produce tropane alkaloids has evolved more than once during the evolution of the angiosperms [127]. Methylecgonone reductase (MecgoR) transcript levels and enzyme activity were analyzed and found to be highest in the young leaves. Immunolocalization experiments show that MecgoR is mainly localized in the palisade and spongy mesophyll tissue of leaves and sepals [151]. Methylecgonone is reduced at the C-3 position to yield methylecgonine. MecgoR is believed to be stereospecific in its reduction of the ketone, yielding exclusively the β-hydroxy isomer. In fact, attempts to use tropine (containing the α-OH) as a substrate in the reverse reaction were unsuccessful. The free β-hydroxy moiety is esterified via a BAHD acyltranferase known as cocaine synthase. This enzyme uses benzoyl CoA as the acyl donor and results in the formation of a benzoyl ester at the C-3 position yielding the final product benzoylmethylecgonine [51]. Cocaine synthase is also capable of using a multitude of alternate acyl-CoA donors, providing an explanation for the presence of alternative tropane alkaloid esters in coca leaves [152,153]. This includes the use of cinnamoyl-CoA to produce the compound cinnamoyl cocaine, a metabolite that can be present at higher levels than cocaine under certain developmental conditions [154]. Immunolocalization of cocaine synthase and cocaine were found to have the highest levels in the palisade layer, however, enzyme and product were also detected at lower levels in the spongy mesophyll and in the upper and lower epidermis [51]. The theoretical tropane alkaloid pathway is presented in Figure 8.

Current molecular data on tropane alkaloid biosynthesis in *Erythroxylum coca* is limited as compared with other known tropane alkaloid producing species [155]. In *Erythroxylum coca*, cocaine and cinnamoylcocaine are stored in the vacuoles of the plants and are complexed with hydroxycinnamoyl quinate esters (HQAs). Chlorogenic acid is the main hydroxycinnamoyl quinate ester responsible for complexing with cocaine and cinnamoylcocaine in the vacuole. A BAHD acyltransferase enzyme (EcHQT) was characterized as the final enzymatic step of hydroxycinnamoyl quinate ester biosynthesis [156].

Plant tissue cultures are useful for biotechnological applications to investigate specialized metabolite pathways due to their facile and scalable nature. Calli tissue cultures are advantageous because, once properly established, they can be maintained indefinitely on solid gel medium by regularly transferring the calli to fresh medium [157]. *E. coca* calli cultures are important to study compounds that can influence and elicit any changes in tropane alkaloid biosynthesis. It has been demonstrated that *E. coca* calli cultures produce cocaine and can be influenced by the type of media the calli tissues are cultivated on, however, known chemical elicitors such as salicylic acid or coronalon had no significant effect on increasing the amount of tropane alkaloids. *E. coca* calli tissue cultures are useful tools to aid in gene discovery and identify unknown enzymatic reactions via stable isotope feeding of known precursors [158]. 

## 7. Perspectives

This new look at the *Erythroxylum* genus and its phytochemicals reveals a mostly untapped, potentially vast interdisciplinary research opportunity that could result in transformative social and economic outcomes. To begin with, *Erythroxylum* species could benefit from the rapid pace of technology development in omics research. This may clarify the evolutionary history and domestication process of species with high cultural significance, while assisting the development of new pharmaceutical, phytotherapeutic, nutritional, and industrial products. For instance, ongoing molecular studies using *Erythroxylum coca* and other relatives are yielding novel genes and enzymes that differ from the previously characterized solanaceous sequences. The potential to modularize these enzymes for metabolic engineering projects shows great promise. Already, studies incorporating both *E. coca* and solanaceous tropane pathway enzymes have been used together in yeast and bacteria to produce the simple tropanes tropine and pseudotropine [159]. Another study also combined several structural genes from both coca and solanaceous species to produce the compound cinnamoyltropine [160]. Indeed, key intermediates such as the *N*-methyl-Δ^1^-pyrrolinium cation have been made in microorganisms suggesting a new system for the manipulation and combination of novel sequences for the purposes of drug design and rapid screening of novel alkaloids [161]. The discovery of convergent evolution in tropane biosynthetic pathways between the Erythroxylaceae and Solanaceae can provide insights into the building blocks of specialized metabolic pathways and how evolutionary pressures have harnessed these blocks to expand chemical diversity in living organisms.

Omics and molecular investigations could be complemented with applied research regarding the safety and value of nutritional, medical, and industrial applications based on coca and other *Erythroxylum* species. A main priority for this research should be the establishment and publicly available release of whole and diverse genomic data from members of the genus, as well as more detailed metabolomic databases both untargeted and targeted towards critical classes of compounds. These resources would then provide a basis for clinical research on coca-derived nutritional, phytotherapeutic and pharmacological metabolites and could help generate novel tools to address pressing public health challenges, such as stimulant addiction, depression, obesity, and malnutrition. Furthermore, these innovations could help strengthen the case for establishing (or strengthening) legal markets for controlled plants like *E. coca* and *E. novogranatense*. This could eventually help shrink the illicit drug trade and its negative effects, while providing licit economic opportunities for populations that are still experiencing the consequences of both illicit markets and harmful drug policies.

## Figures and Tables

**Table 1:** Bioactive properties within some members of the genus *Erythroxylum.*

Species	Distribution	Type of Study	Bioactive Properties	Extract Source	Active Compounds	References
*Erythroxylum vacciniifolium* Mart.	Brazilian northeast, Atlantic Forest	Pre-clinical testing: Lymphotropic virus type I (HTLV-1) positive MT-4 cells and mice	- Aphrodisiac - Tonic - Antimicrobial - Anticancer	Stem bark	C-3 α ester; C-3 3,4,5 trimethoxybenzoic acid; pyrrole-2-carboxylic acid; cinchonains 1a and 1b	Zanolari et al. (2003) Graf et al. (1978) Manabe et al. (1992) Satoh et al. (2000)
*Erythroxylum ovalifolium* Peyr.	Restinga (sandbanks) in the state of Rio de Janeiro (Brazil)	Pre-clinical testing: Swiss mice	- Neutralize toxicity of snake venom - Treat edemas and hemorrhages - Anti-fungal	Stem bark	Friedelin and Lupeol	Coriolano de Olivero et al. (2016)
*Erythroxylum pervillei* Baill.	Endemic to Madagascar	Testing: Human ovarian adenocarcinoma (SKVLB) cells and multidrug-resistance oral epidermoid carcinoma (KB-V1) cells	- Anticancer - Treat abdominal pain	Stem bark and roots	- Previlleine A, G and H - Aromtic sters	Chin et al. (2006) Silva et al. (2001)
*Erythroxylum macrocarpum* O.E. Schulz	Endemic to Mauritius	Pre-clinical testing: Swis albino rats	- Antibacterial - Diuretic	Leaves and twigs	- Tannins - Flavonoids - Tropan-3α-ol - tropan-3β-ol - 6β-diol	Mahomoodally et al. (2005) Al-said et al. (1986)
*Erythroxylum caatingae* Plowman	Dry forest in northeastern Brazil known as Caatinga	Pre-clinical testing: Swiss mice and human cancer cells from leukemia (K562), lung (NCI-H292) and larynx (Hep-2)	- Anticancer - Antimicrobial	Stem	- 6β-Benzoyloxy-3α-(3,4,5-trimethoxybenzoyloxy)	Aguiar et al. (2012)
*Erythroxylum suberosum* A.St.-Hill., A.Juss & Cambess.	Savannahs in Brazil, Bolivia, Paraguay, Venezuela and the Guyanas.	Testing: Human cancer cells of oral squamous carcinoma (SCC-9), hypopharynx squamous carcinoma (FaDu) and human keratinocyte (HaCaT)	- Antidiarrhea - Astringent - Antirheumatoid - Anesthetic - Antioxidant	Leaves	- Coumarins - Flavonoids - Isoquercitrin - Catechin	Riberio et al. (2015) Barros et al. (2017) Macedo et al. (2016)
*Erythroxylum laurifolium* Lam.	Endemic to Mauritius	Testing: Kidney epithelial cells (VERO)	- Anti-diabetic - Anti-hypertension - Effects against Herpes I virus	Leaves	- Afzelin - Quercitrin - Tannins - Flavonoids	Picot et al. (2014) Hansen et al. (1996) Lohezic et al. (1999) Jelager et al. (1998)
